# Systematic Literature Review of Health Impact Assessments in Low and Middle-Income Countries

**DOI:** 10.3390/ijerph16112018

**Published:** 2019-06-06

**Authors:** Meelan Thondoo, David Rojas-Rueda, Joyeeta Gupta, Daniel H. de Vries, Mark J. Nieuwenhuijsen

**Affiliations:** 1 Barcelona Institute for Global Health (ISGlobal), Centre for Research in Environmental Epidemiology (CREAL), 08003 Barcelona, Spain; david.rojas@colostate.edu; 2 Amsterdam Institute for Social Science Research (AISSR), University of Amsterdam, 1018 WV Amsterdam, The Netherlands; j.gupta@uva.nl (J.G.); d.h.devries@uva.nl (D.H.d.V.); 3 Department of Environmental and Radiological Health Sciences, Colorado State University, Fort Collins, CO 80523, USA; 4 Faculty of Medicine and Health Sciences, University of Barcelona (UB), 08036 Barcelona, Spain; 5 Department of Biomedicine, University Pompeu Fabra (UPF), 08005 Barcelona, Spain; 6 Department of Environmental Epidemiology, Municipal Institute of Medical Research (IMIM-Hospital del Mar), 08003 Barcelona, Spain; 7 Department of Epidemiology and Public Health, CIBER Epidemiología y Salud Pública (CIBERESP), 28029 Madrid, Spain

**Keywords:** health impact assessment (HIA), systematic review, process evaluation, low and middle-income country

## Abstract

Health Impact Assessments (HIAs) motivate effective measures for safeguarding public health. There is consensus that HIAs in low and middle-income countries (LMICs) are lacking, but no study systematically focuses on those that have been successfully conducted across all regions of the world, nor do they highlight factors that may enable or hinder their implementation. Our objectives are to (1) systematically review, geographically map, and characterize HIA activity in LMICs; and (2) apply a process evaluation method to identify factors which are important to improve HIA implementation in LMICs. A systematic review of peer-reviewed HIAs in 156 LMICs was performed in Scopus, Medline, Web of Science, Sociological abstracts, and LILACs (Latin American and Caribbean Health Sciences) databases. The search used PRISMA (Preferred Reporting Items for Systematic Reviews and Meta-Analyses) guidelines and covered HIAs across all type of interventions, topics, and health outcomes. HIAs were included if they reported a clear intervention and health outcome to be assessed. No time restriction was applied, and grey literature was not included. The eligible studies were subjected to six process evaluation criteria. The search yielded 3178 hits and 57 studies were retained. HIAs were conducted in 26 out of 156 countries. There was an unequal distribution of HIAs across regions and within LMICs countries. The leading topics of HIA in LMICs were air pollution, development projects, and urban transport planning. Most of the HIAs reported quantitative approaches (72%), focused on air pollution (46%), appraised policies (60%), and were conducted at the city level (36%). The process evaluation showed important variations in the way HIAs have been conducted and low uniformity in the reporting of six criteria. No study reported the time, money, and staff used to perform HIAs. Only 12% of HIAs were based on participatory approaches; 92% of HIAs considered multiple outcomes; and 61% of HIAs provided recommendations and fostered cross-national collaboration. The limited transparency in process, weak participation, and inconsistent delivery of recommendations were potential limitations to HIA implementation in low and middle-income countries. Scaling and improving HIA implementation in low and middle-income countries in the upcoming years will depend on expanding geographically by increasing HIA governance, adapting models and tools in quantitative methods, and adopting better reporting practices.

## 1. Introduction

In the last 30 years, Health Impact Assessments (HIA) have been promoted as a key instrument to safeguard public health [1,2]. HIAs combine mixed-methods to systematically judge the potential health effects a proposed policy, program, or project might have on population health and the distribution of those effects within a population [3]. HIAs are useful to predict the impact of interventions (interventions are defined as either policy, program, or project in this paper) in shaping health determinants before they are framed and implemented. They have been promoted as an important tool to achieve health equity. HIAs have been successfully and extensively used in cities of high income countries (HICs) to assess the impacts of air pollution [4], urban planning [5], and transport [6,7]. Yet, their implementation at the global level remains hampered by the disparity in practice between high and low and middle-income countries (LMICs), also referred to as low resource countries in this paper [8].

There is more scientific understanding on the potential rather than implementation of HIAs in low and middle-income countries (LMICs). Literature examining HIAs in LMICs has focused on gaps in policy rather than gaps in practice [9,10]. Evidence shows that compared to HICs, very few LMICs have regulatory policy frameworks on HIA. In some Asian countries, HIA legislation at national and subnational level exists. Thailand has institutionalized HIA in its Constitution, while Laos, Cambodia, and Malaysia have integrated HIA as part of the Environmental Impact Assessment (EIA) processes [10]. Vietnam is in the process of incorporating the HIA framework in its Health Action Plan [11]. In Latin America, only Mexico and Brazil have published national-level guidelines on HIA [12]. No African country actively promotes or regulates HIA [10,13]. While the presence of firm policy frameworks is a major requirement for HIA, it does not necessarily imply that one country is more effective in implementing HIA than another [14].

Understanding and addressing barriers to HIA in LMICs is imperative for ensuring equity in HIA practice across the globe. The value for equity weighs even more so as low and middle-income countries absorb an unequal burden of health impacts generated from accelerated environmental anthropogenic changes. Compared to HICs, LMICs are disproportionately exposed to modern health hazards such as water, urban air and noise pollution, deforestation, land degradation, waste management, and climate change [15]. Most of the 7 million people (92%) dying from exposure to air pollution across the globe live in LMICs. The same countries also claim 90% of 1.25 million traffic-related deaths and 80% of 56.9 million deaths caused by non-communicable diseases, per year, in the world [16,17,18,19]. Yet, Erlanger et al. identified that only 6% of all HIA publications were conducted in LMICs [8]. HIA is an uncommon and inconsistent practice in Latin American and Caribbean countries (LACs) [20] where it is limited to approval mechanisms for privately-led projects [21,22]. Other studies confirm that the focus on private rather than public projects also drives HIA in Africa [23,24]. Such trends stand in stark contrast with the consistent and mostly regularised HIA practice in high income countries. Reviews focusing on HIA in the USA [25], Europe [26,27], Australia [28,29], and New Zealand [30] show that HIAs in HICs focus on diverse topics, are used in both public and private realms, are led by varied institutions and professionals, and apply different types of quantitative and qualitative methods to calculate health outcomes. 

To our knowledge no review has addressed detailed HIA trends in LMICs. While some reviews [8,9,10,12,31,32,33,34,35,36] have reached consensus that HIAs in low resource countries are lacking, there is no systematic review of case studies that have been successfully conducted in LMICs and there is very little understanding of how they were conducted. As far as we know, no systematic method or process evaluation assessment has been used to define exactly where and how HIAs are being conducted, by whom, and for what purpose, in LMICs across all regions of the world.

Process evaluations provide information on why and how HIAs are conducted [37]; they are useful to determine ways for improving methods and expanding HIA practice, but so far, they have been completed in high income countries only [38,39,40,41]. Hence, this study had two objectives. First, we performed a systematic literature review of HIAs to identify and audit HIA activity across LMIC geographical settings. Second, we conducted a process evaluation assessment based on six criteria to identify factors that enabled or hindered implementation of HIA in LMICs. The process evaluation addressed the ‘how’ aspects of HIA case studies (who conducted, on what topic, where, which outcomes, stakeholders involved, when, etc.) via research questions and by reporting issues across eligible peer-reviewed papers only. Due to the scarcity of cases per country and lack of rigorous methods to assess HIA impact [42], we did not address the ‘why’ aspects (impact evaluation) of HIA, and we state the limitations of our approach in Section 4.3 and Section 4.5.

## 2. Materials and Methods

### 2.1. Systematic Review

A systematic review method was used, complying with the Preferred Reporting Items for Systematic Review and Meta-Analysis Protocols (PRISMA-P) [43] (see Supplementary File 1). The systematic review has been registered in the PROSPERO database (registration number: crd420118102715) since 8 August 2018. PROSPERO is an international database of prospectively registered systematic reviews in health and social care (see http://www.prisma-statement.org/Protocols/Registration). Articles were systematically screened from five online databases—Scopus, Medline, Web of Science, Sociological abstracts, and LILACs—from inception until 13 May 2018. In total, the review included 156 low and middle-income countries, classified as ‘Emerging Market and Developing Economies’ (EMDE) in the World Economic Outlook 2016 [44] and referred to as ‘LMICs’ for the purpose of this paper.

**Figure 1:**
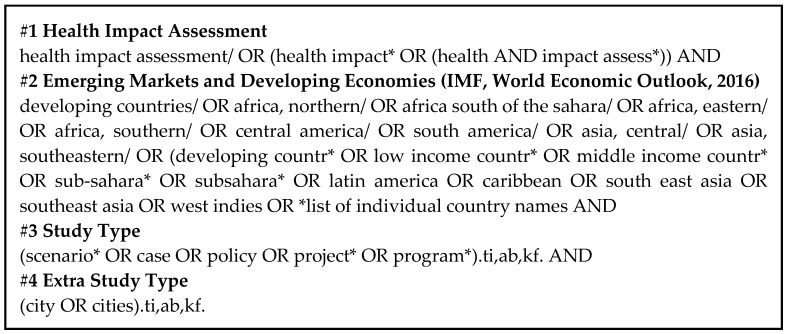
Search string used for the systematic review.

The review included standalone HIA case studies (original articles) conducted in low and middle-income countries and published in peer-reviewed papers. General articles discussing the state-of-the-art of HIA, methodological concerns, as well as opinion papers were not considered. No time restriction was applied, and grey literature was not included. The search was conducted in English, Portuguese, French, and Spanish. The search string combined #health impact assessment, #country, #study, type, and #city specifier (see Figure 1). The city specifier was added in order to identify city-level HIAs that may not have mentioned national level proposals or approaches. To ensure the thoroughness of peer-reviewed studies, additional records were identified via manual sources: a manual bibliographic review (checking reference lists of selected papers), internet searches, and expert consultation. Two independent researchers (M.T. and D.R.R.) performed all levels of screening and resolved discrepancies by consensus.

#### 2.1.1. Eligibility Criteria

HIAs were included if they reported a clear intervention and health outcome to be assessed. Additionally, case studies were included if:

1. The appraisal provided a comparison between different situations and brought an assessment that would change the status quo.
2. There was a clear statement and description of an intervention to be assessed. The intervention could be a program, project, or policy.
3. The intervention triggered a ‘before and after’ situation: It reported a change in the distribution of exposure for at least one health pathway.
4. The intervention addressed one or more problems in a specified population: It reported a change in at least one health outcome.

#### 2.1.2. Data Extraction

We extracted data from eligible studies using an Excel-based extraction tool (Supplementary File 2) split in two parts: general characteristics and process evaluation assessment. The general characteristics enabled a descriptive analysis of HIA case studies: author, title, year of publication, country, level at which conducted, type of object appraised, data type used, self-identification as HIA, topic of HIA. The process evaluation assessment consisted of six process evaluation criteria justified in the extraction tool.

#### 2.1.3. Process Evaluation Assessment

We conducted the process evaluation assessment by selecting and adapting five questions from Quigley and Taylor (2004) [37]:

1. What data were used and what types of outcomes were calculated?
2. What resources (financial, human, time) were needed to complete the HIA?
3. Who and how were different stakeholders involved and engaged in the process?
4. How and when were the recommendations delivered to the relevant decision makers?
5. What collaborations existed that led to the publication of the HIA?

We then searched for the process evaluation criteria most likely to respond to the previous questions by reviewing HIA methodological literature [34,45,46,47,48,49,50,51,52,53] and existing reviews [8,9,10,12,31,32,33,34,36]. Based on this non-systematic review, we defined six evaluation criteria: (1) access to baseline local data; (2) resources used; (3) based on participatory approaches; (4) consider multiple outcomes; (5) provide recommendation; and (6) foster cross-national collaboration) (see Table 1). In regards to the last criteria, shared authorship and first author affiliation to a local institution were considered as a research output on HIA from the local country. The affiliations of each collaborating author were not detailed; however. the presence of shared authorship with a foreign institution was checked for. The presence of foreign collaboration is reported as an existing recommendation for HIAs to build cross-national scientific ties that in turn encourage the increase and expansion of HIA implementation [54]. A series of associated factors were inductively generated and systematically applied to all case studies. The reporting or non-reporting of each criteria were useful to identify factors enabling or hindering implementation of HIAs in LMICs. 

## 3. Results

**Figure 2:**
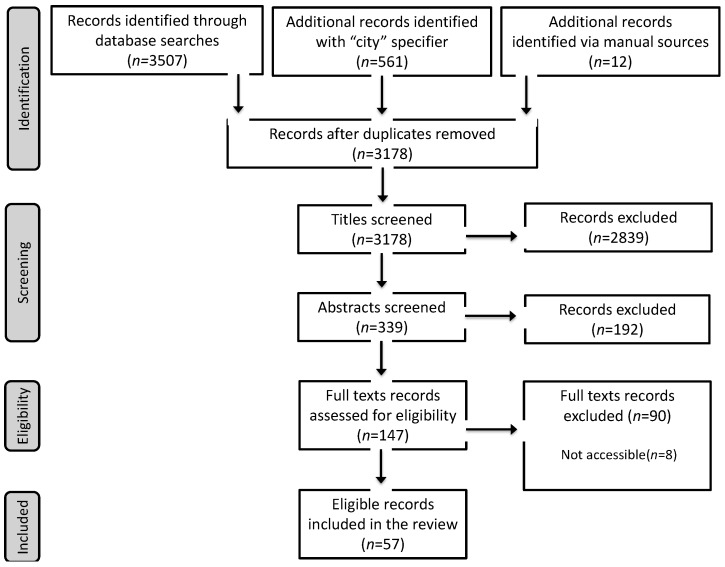
Study selection flowchart.

Our search yielded 3178 records initially (excluding 902 duplicates). After title screening (retaining 339 records) and abstract screening (resulting in 147 studies), we conducted a full-text eligibility assessment and discarded 90 records not satisfying the inclusion criteria. The final dataset included 57 studies (see Figure 2 for PRISMA flowchart and Supplementary File 3 for the list of studies). We present the results as follows. We first describe the general characteristics of HIAs in LMICs. We then specify the geographic and regional distribution of HIAs. Finally, we report on each process evaluation criteria separately. 

### 3.1. HIA General Characteristics

The eligible papers are dated from 1997 to 2018, of which 75% (*n* = 40) were published after 2010. A larger number of HIAs were conducted at city levels (*n* = 21) as opposed to national (*n* = 15), sub-national (*n* = 11), project (*n* = 7), regional (*n* = 2), and global (*n* = 1) levels. Sub-national HIAs included both urban and rural HIAs. More HIAs were used to estimate the effects of policies (*n* = 34) rather than programs (*n* = 12) or projects (*n* = 11). Quantitative HIAs were the most common, covering 72% of cases (*n* = 41). The remaining cases were split evenly in mixed-methods and qualitative HIAs (each *n* = 8). Only 30% of cases self-identified as HIAs by either defining HIA itself or clearly describing the stages used to perform HIA. Other cases defined themselves as epidemiological or health risk assessment studies.

### 3.2. Geographic Distribution and Affiliation

HIAs were conducted in 26 of the 156 countries reviewed (16%). They were unevenly distributed across regions: Asia (46%, *n* = 25), Africa (18%, *n* = 10), Europe (18%, *n* = 10), and Americas (16%, *n* = 9). All single-country studies were conducted in the Global South except for one completed in Hungary [55]. The number of HIAs varied across countries, with the highest number of HIAs conducted in China (see Table 2). HIA topics also differed across regions, with Asia leading on the wider diversity of topics: 9 in total. In contrast, Africa, Europe/Middle East, and Americas covered 5, 6, and 5 topic categories, respectively (see Table 3). Air pollution (*n* = 26) is the most common, and it is the only topic for HIA that spread across all regions and a larger number of countries. Out of the 10 studies conducted in Africa, half focused on development projects. To date, Africa is the only region where no HIAs on urban transport planning have been published. By order of importance, the three leading topics for HIA in LMICs (number of times it occurred in the data) were air pollution, development projects, and urban transport planning (see Table 4). Overall, the first author was affiliated to a local institution in 49% of cases (*n* = 27). First author affiliation varied across continents: In Africa, it involved 20% of cases (*n* = 2/10), versus 42% in Asia (*n* = 11/25), 56% in the Americas (*n* = 5/9), and 90% in the Europe/Middle East region (*n* = 9/10). 

### 3.3. Results from the Process Evaluation

The process evaluation shows important variations in the way that HIAs were conducted, and there is low uniformity in the reporting of the six process evaluation criteria (see Table 5).

#### 3.3.1. Access to Baseline Local Data

All HIA studies accessed local baseline data to estimate health impacts, of which 75% (*n* = 43) used existing baseline datasets. Via the assessment, access to data was not reported as problematic; however, studies reported the lack of quality in baseline data of quantitative HIAs as a major limitation. Several quantitative HIAs reported that using weak quality datasets made it difficult to estimate accurate differences among cities, variations in emission scenarios, and changes in population distributions [56,75,82,86,101]. Even when primary datasets were collected from scratch (25%, *n* = 14), important assumptions on data validity had to be made [89]. In Bejaia for instance, Benaissa et al. collected data on exposure to particulate matter (PM_10_) but had to assume that estimates remained constant despite seasonal variations [88]. Kahn et al. had to complement local data with disease data from neighboring Uganda to estimate the impact of a multi-disease prevention campaign in Kenya [90]. Mestl et al. used data from Bangladesh to estimate indoor air pollution impacts in a rural area of China [67]. Furthermore, the treatment of local quantitative datasets using non-local dose-response functions or incidence rates were reported to skew HIA outcomes because they were not applicable for different levels of exposures, local population sensitivity, and age distribution. Studies in Chile [111], China [56], Peru [101], Brazil [75], Iran [83], and Turkey [86] show that consequences include the underestimation of health effects, limitations to primary (rather than secondary) impact assessments of indicators, and restriction in the selection of health endpoints.

#### 3.3.2. Reporting Resources Used

No study reported the time, money, and staff used to perform HIA. The lack of information on the resources used for the studies made it difficult to assess what is needed in terms of cost, time, and human resources to conduct HIA in a lower resource setting. 

#### 3.3.3. Based on Participatory Approaches

Only 12% (*n* = 7) of HIAs were based on participatory approaches, all of which were either mixed-methods or qualitative HIAs. No quantitative HIA reported stakeholder participation. All participatory-based HIAs provided the stage at which participatory activity occurred and described the profile of stakeholders involved in the participation [81,91,96,104,112,113,114]. The participatory approaches in the process of HIA was unclear in 50 studies, i.e., 87% of cases did not conduct or report participatory activities. It is unclear if and why stakeholders were not effectively engaged in HIAs. However, wherever present, authors reported that stakeholder participation was valuable to set the boundaries of the assessment [96], to clarify expectations and disaggregate different determinants of health [112], and to identify and concretize collaboration [113]. Participation was conducted via qualitative interviews, focus group discussions, and during field visits at screening and scoping stages [91,96,104,112,113] or at reporting stages [81]. Only one study reported participation (consulting for stakeholder feedback) after the HIA was conducted [112].

#### 3.3.4. Considered Multiple Outcomes

92% of HIAs considered multiple outcomes. Morbidity outcomes were calculated in 75% (*n* = 43) of studies across 25 countries. Mortality outcomes were calculated in 58% (*n* = 33) of studies across 15 countries. Cost outcomes were calculated in 31% (*n* = 18) of studies across 11 countries and social determinant outcomes in 11% (*n* = 11) of studies across 13 countries (see Table 6). Although Brazilian scholars reported that calculating mortality outcomes remains the best choice (more robust and of high quality) in a city like Sao Paolo [75], the majority of studies (92%, *n* = 53) assessed at least two or more health impact outcomes (including mortality) and reported benefits of considering multiple outcomes. Only one study calculated all four outcomes simultaneously [101]. 

Some examples of morbidity outcomes were respiratory and cardiac hospitalizations in Algeria [88]; total mortality, cardiovascular mortality, respiratory mortality, respiratory disease, hospital admission, and cardiovascular disease in Iran [83]; avoidable traffic deaths in China [115]; and HIV (Human Immunodeficiency Virus) cases in Chad-Cameroon [23]. Examples of cost outcomes were calculated economic loss (as a share of Thailand’s Gross Domestic Product) due to exposure to PM_10_ emissions from transportation [73], and in monetary terms of health benefits (less mortality and less hospitalizations) of the flue-gas desulphurization units of a coal-fired power plant in Turkey [85]. Studies show that aiming for different outcomes encourages cross-sectorial and transdisciplinary work. A HIA conducted in Bangladesh showed that estimating the health impacts of brick construction should be accompanied by an assessment of social and labor issues [92]. In India, a multilateral sectoral approach combining mining and transport was adopted to estimate health impacts of air pollution (particulate matter 10), resulting in wider options for risks mitigation involving energy efficiency, cleaner technology, and enforcement of control policies [80]. 

#### 3.3.5. Provided Recommendations

In general, 63% (*n* = 43) of HIAs provided recommendations. However, the delivery of recommendations was inconsistent, ranging from HIAs providing specific, brief, or no recommendations at all. Specific recommendation sections were found in 16% (*n* = 9) of studies. Brief recommendations incorporated in the conclusion section were reported in 56% (*n* = 33) of studies in the format of one-sentence (*n* = 7) and less-than-one-paragraph (*n* = 26). Two studies reported that separate reports targeted to specific stakeholders were generated from the HIA (these papers were counted as having separate recommendation sections) [106,114]. The time at which recommendations were delivered also varied within the 14% (*n* = 8) that provided such indication: early (*n* = 2), mid (*n* = 0), and later stages (*n* = 6). More than half of the studies not providing recommendations (60%, *n* = 9/14) were conducted on air pollution.

#### 3.3.6. Fostered Cross-National Collaborations 

The opportunity to foster cross-national scientific collaborations was reflected in the fact that more than half of the HIAs were published by teams based in different countries. Indeed, evaluating shared authorship showed that 61% (*n* = 35) of HIAs were published jointly by local and foreign researchers. The remaining HIAs were published exclusively by local teams (32%, *n* = 18) or by foreign teams (10%, *n* = 6). Foreign-led HIAs (HIAs led by non-local teams) were either published by small teams of one or two authors [23,102] or by larger teams working on quantitative HIAs in China [59,65,67] and Thailand [73]. Locally-led HIAs were conducted in Brazil, China, India, Iran, Thailand, Turkey, and Mexico [9,10,12,57,116,117], but other HIA studies within the same countries also showcased local-foreign co-authorships. In this regard, Chinese case studies stood out. Six out of 15 studies were published by first authors affiliated with Chinese academic or research institutes. The 9 remaining studies were published by first authors affiliated with academic or research institutes in Norway, Netherlands, Germany, Switzerland, UK, USA, and Belgium, of which 3 studies included no China-based authors.

## 4. Discussion

Peer-reviewed HIAs were conducted in 26 of 156 LMICs (16%) and were unevenly distributed across regions. A larger number of HIAs used quantitative approaches, focused on air pollution, appraised the effect of policies, and were conducted at the city level. The process evaluation shows important variations in the way that HIAs are conducted and low uniformity in the reporting of six process evaluation criteria. This study fills an important gap by mapping, comparing, and critically evaluating HIAs conducted in LMICs. It uses empirical evidence reported by HIA case studies and adds value to rare studies that attempt to examine HIA activity in developing regions of the world [8,10,12,14]. The study provides solid baseline information about the characteristics of HIAs and their limitations. The search selection bias of case studies was reduced by combining databases from different regions and fields. Time restrictions were removed and language barriers reduced. Adopting a systematic search strategy with wide inclusion criteria (see Supplementary File 1) was also efficient for ensuring all relevant scientific evidence on the topic was gathered.

### 4.1. Geographic Scaling

This review showcases the inequitable distribution of HIAs among low and middle-income countries of the world, reasonably questioning the role of equity as one of the four ground values of HIA practice [1]. Geographic scaling is one solution to address this imbalance; however, the consideration of factors justifying weak implementation in these regions is crucial. Scholars have previously identified the lack of simplified tools, inadequate policy guidelines, poor governance, weak capacity, no solid environmental baseline databases, and lack of scientific collaboration as barriers to scaling HIA practice [10,14,118]. Our findings, however, indicate that local teams are initiating and completing HIA processes despite these vast challenges by adapting traditional methods of data collection and analysis. 

Existing academic work showed that in 2005, quantification was comparatively rare in HIA [33], which contrasts with our current findings that a significant share of HIAs in LMICs were quantitative. Studies reported that the use of quantitative methods in geographical settings where datasets were of weak quality called for adapted solutions. In our review, studies showed alternative avenues for impact modelling, for instance, to make up for the lack of incidence data [86,97]. In qualitative HIAs, different data collection methods were employed, including the use of participatory approaches such as stakeholder e-interviews [112], and news virtual tools such as Google Earth were applied [119]. In existing literature, both Abah (2011) and Winkler et. al (2012) have suggested that complementing existing datasets with newly conducted, comprehensive health surveys and cross-sectional studies can compensate for the lack of reliable data [120,121]. Particularly, Winkler et al. (2012) encourage the use of tools such as the gap analysis to assess availability as well as quality of existing data before deciding to do a HIA [121]. Other scholars have argued the importance of strengthening local governance structures and policy frameworks to facilitate HIA practice in challenging contexts [2,120,122].

Interestingly, our findings show that countries where at least two HIAs were conducted are the same ones known to host some form of HIA legislation, regulation, or framework. Such are the cases of Brazil, China, India, Iran, Thailand, and Turkey [9,10,12,57]. Additionally, this exact set of countries, adding Mexico, corresponded to where HIAs were conducted and published by exclusively local teams (no foreign teams were mentioned in authorship or acknowledgements), suggesting some level of local governance as well as the presence of a technical and resource capacity at the country-level.

In addition to the presence of policy frameworks, Joffe and Mindell (2002) suggested that focusing on the right HIA sectors (i.e., those most affecting health) would lead to HIAs that have significant scientific, environmental, social, and political relevance [123]. They suggested that HIAs should focus on the sectors most urging for assessments in LMICs: transport, nutrition, and housing [123]. Yet, in the 10 studies of Africa alone, none of these are touched upon; focus has been cast, rather, on air pollution, waste, dam and mining projects, homosexuality bills, and infectious diseases [23,89,90,91,93,95,104,105,106,124]. This suggests that the proposal made by Joffe and Mindell (2002) is either outdated or not adapted to LMICs. In contrast, our review shows that exposure to air pollution is the only area of focus assessed in all four regions and by the most amount of countries. Our findings show that LMICs have a significant interest in the topic of development projects, which other authors have justified previously [22,125,126]. However, despite the increasing amount of road-related deaths in Africa [127], our findings show that Africa is the only region where no HIAs on urban and transport planning has been published so far.

### 4.2. Methods

HIAs showed significant differences in the application of process evaluation criteria. Similar variations were identified in the USA [128], indicating that strict compliance to guidelines and standards may be a luxury that HIA teams worldwide find challenging to afford. Additionally, the diversity in process confirms that the criteria and pre-requisites for ensuring effective HIA implementation can be difficult to define [53,129]. This challenges the idea that a set of core universal principles can ensure the effectiveness of HIA as suggested by Fakhri et al. [52], because the level of compliance with a set of standards is not necessarily representative of effectiveness or quality [29].

The criteria assessing to what extent HIAs are based on participatory approaches is a good example illustrating that process criteria may be, but are not necessarily, reflexive of effectiveness or quality. Our review shows that participatory approaches were reported exclusively in qualitative or mixed-methods studies. Yet, it is difficult to assess whether that means that they are of better quality or higher effectiveness than quantitative studies not reporting participatory approaches. Current literature urgently calls for quantitative HIAs to integrate participatory approaches as part of their frameworks [130]. Benefits include the involvement of communities most affected by projects, programs, or policies; inducing stakeholder engagement at different levels of actions; increasing public acceptability of interventions; and tackling complex issues of the urban realm. While several papers confirm the benefits of involving different profiles of participants in the physical vicinity of projects [24,114] with particular ethnic backgrounds [76] or with specific expertise [93], there is not enough information to assess the quality of the participatory approaches used. This confirms recent findings that HIA authors need to use more rigorous methods when conducting and reporting participatory approaches such as sampling methods, time and scale of participation, and objectives, etc. [32].

The lack of recommendations emerged as a major methodological problem in this review. Literature supports that bad delivery or report of recommendations influence the integration of HIA in policy making [40,131,132,133]. Davenport et al. (2005) found that providing realistic and non-controversial recommendations, fitting in political timeframes, are important enablers to the integration of HIA findings into the decision-making process [40].Harris et al. (2014) go further by stating that adequate recommendations define whether HIA becomes relevant and absorbed into policy decision-making [133]. Even further, authors of a previous HIA evaluation study excluded upfront cases with no clear recommendations and considered the latter a pre-requisite to scientifically relevant HIAs [29].

We were therefore surprised to observe that no recommendations were formulated in 26% (*n* = 14) of studies. The inconsistency in the timing and format of delivery made it hard to assess if recommendations effectively led to evidence-based policy actions. However, studies presenting a separate and specific section with recommendations provided more insight on the policy implications of their findings [60,93,103,134] and provided information on the expert panel towards whom the recommendation report was aimed at [23]. Other practical and action-oriented recommendations were provided for a dam in Zimbabwe [105], a mosquito-borne program in India [81], a homosexuality bill in Uganda [104], and a mining project in the Democratic Republic of Congo [95].

### 4.3. Reporting

A major consequence of bad reporting is a serious lack of transparency in the methods and the difficulty in detecting HIAs upfront. Our experience confirms that HIAs can be very difficult to identify because there is no single framework or detailed checklist procedure to qualify what actually constitutes a HIA [135]. The lack of definition and clarity of what processes were adopted significantly challenged the identification and comparison of HIA processes across settings. This was aggravated by the low percentage of cases self-identifying as HIAs (either by defining HIA itself or clearly describing the stages used to perform HIA) to start with. Some authors declared having done HIA without referring to any HIA standard guideline or standards [136] and were excluded. Others performed HIA without claiming or defining it as such [56,102], and the term HIA was not always used in the same sense across studies. The lack of definition and transparency in HIA processes that came from studies in China, Turkey, and Mongolia [70,87,100] were harder to identify and include; they could have been discarded due to close similarities with health risk assessments (HRAs). HRAs are an integral part of HIAs (often conducted in the appraisal stage) but are not HIAs. Risk assessments could estimate the effects of a particular exposure/risk/danger but do not always assess the impact of a particular change in the current situation due to a clearly stated intervention. The most easily identifiable and analyzable HIA cases described the type of HIA conducted, the data collection approach, and clearly identified the basic procedural stages. Some examples included, but were not limited to, a study in Kenya assessing the impacts of a dam and irrigation projects [91] and a study assessing housing policies in central Europe [76].

No study reported the resources needed to conduct a HIA, limiting our ability to assess what resources could be considered sufficient to successfully complete the process. Having a better idea of such elements is crucial to justify the cost- and resource-effectiveness of HIAs in low and middle-income countries. In HICs, benefits of HIAs have been proven to outweigh the cost of undertaking them and not the contrary [41]. However, evidence shows that policy makers decline HIA use because they incorrectly believe that HIAs are ‘expensive and time-consuming’, both in HICs and LMICs [29,137]. Earlier, Kemm (2005) reported the need of conducting cost-benefit analysis of HIA as an important element and low-hanging fruit for progress [134]. Other authors have also flagged the lack of information on HIA costs but none address LMICs specifically [135,136]. It is crucial to start assessing and reporting the cost of HIAs in LMICs to increase policy dialogue around institutionalization of HIA, not only for the sake of awareness but also to enable to assessment of what benefits actually exist and at what cost.

### 4.4. Recommendations

Based on the empirical review of 57 HIA case studies from LMICs, we provide a simplified “Process Appraisal Checklist” adapted from Parry and Kemm’s criteria for process evaluation (2005) [48] (see Table 7). We adapted the challenges and opportunities identified during the process evaluation and adapted them to each stage of the existing checklist in order to provide more practical guidelines for scholars or professionals interested in conducting HIA in LMICs.

Based on the reporting of process evaluation factors, we also propose the following recommendations:

1. For quantitative HIAs, assess the data availability and quality at screening and scoping stages so as to plan in advance for solutions to tackle inadequate baseline datasets (either no, insufficient, or bad data). In LMICs, both availability and quality of data should dictate whether a HIA is conducted or not; after which HIA frameworks need to be adapted to what can be done with the resources (human, financial, and time) at hand. A thorough understanding of HIA typologies (see Harris-Roxas (2011)) [137] can be helpful to identify the type of HIA most fitting for conducting a quality HIA with available data. For instance, the choice of running a rapid, intermediate or comprehensive HIA can significantly influence the scope, impact, and ultimately the action taken upon HIA estimates.
2. The use and accurate reporting of participatory approaches is encouraged for all types of HIA, including quantitative HIAs.
3. HIA practitioners should ensure that clear recommendations are formulated from the HIA outcomes. Such recommendations should be well-framed and delivered with adequate timing and to the right people.
4. Adopt a transparent process by reporting the staff, cost, time, and training needed to conduct the HIA. This will facilitate knowledge transfer of good practices and comparative studies across countries.
5. Engage into collaboration at local, regional, and international levels. Local collaboration between sectors and institutions is as important as cross-national collaborations for building awareness and increasing technical capacity in the country.
6. Plan for the evaluation of successfully conducted HIAs in order to ensure quality and assess the cost-effectiveness of the process.

### 4.5. Study Limitations

Despite a solid systematic search, all relevant studies may not have been identified. The exclusion of grey literature may have induced publication bias as HIA in lower resource countries is frequently conducted by private or multilateral organizations [23,113,138]. It is also possible that HIAs driven for specific interventions on controversial topics and within tighter timelines were not made public or are restricted for use by particular individuals or institutions. Studies with negative findings, bad experiences, or that were unsatisfactorily completed may have been less likely published. The exclusion of non-Latin languages such as Chinese may have excluded some studies. As another limitation, the process evaluation criteria were selected according to the authors’ professional judgement and may have impacted on the findings. Furthermore, many published HIAs are not required to include any of those criteria, and even if they did, they may not have reported it, especially if publication space is restricted. The interpretation of evidence must also be done with care as they are mostly based on the subjective assessment of authors. While factors such as outcome calculation, regional distribution and level of implementation are objective to assess, the interpretation of other factors such as participation and recommendation were less evident. For instance, it was difficult to assess whether the absence of participation and recommendation were due to lack of reporting or lack of accomplishment.

## 5. Conclusions

The systematic review with focus on process evaluation of 57 case studies provided a unique opportunity for mapping and assessing HIA activity in LMICs. There is an unequal distribution of HIAs in LMICs. Studies from Asia spearhead in number and diversity of topics. The leading topics of HIA in LMICs are air pollution, development projects, and urban transport planning. Studies in Africa are significantly lagging behind in terms of first author affiliation. The process evaluation showed important variations in the way HIAs are conducted and low uniformity in the reporting of the six criteria. The limited reporting of resources used, weak participatory approaches, and inconsistent delivery of recommendations were potential limitations to scaling HIA practice in LMICs, while current opportunities to scaling HIAs are driven by access to local baseline data, the consideration of multiple outcomes, and strong cross-national collaborations. Finally, the potential for scaling HIA to low and middle-income countries over the upcoming years will depend on adapting quantitative methods to data availability and quality, adopting better reporting practices, and pushing for policy frameworks that promote HIA, especially in countries where it is most needed.

## Figures and Tables

**Table 1:** Criteria and associated factors for process evaluation.

Question No.	Criteria	Associated Factors	Description
1	Accessed baseline local data	1.1. Use of existing database 1.2. Primary data collection	Yes or no Yes or no
2	Reported resources used	2.1. Human 2.2. Time 2.3. Money	Yes or no Yes or no Yes or no
3	Based on participatory approaches	3.1. Stage of participation activity 3.2. Participant profile described	Screening, scoping, etc. Yes or no
1	Considered multiple outcomes	4.1. Multiple outcomes 4.2. Coverage per outcome	Yes or no By mortality, morbidity, cost, social outcomes
4	Provided recommendation	5.1. Format 5.2. Timing of delivery	Brief, separate section Early, mid and later stages
5	Fostered cross-national collaboration	6.1. Shared authorship (local & foreign) 6.2. Local affiliation of first author	Yes or no Yes or no

**Table 2:** Number of studies by country.

**Single-Country Location**	**Number of HIAs**	**Reference**
China	15	[56,57,58,59,60,61,62,63,64,65,66,67,68,69,70]
Thailand	4	[71,72,73,74]
Brazil	4	[75,76,77,78]
India	3	[79,80,81]
Iran	3	[82,83,84]
Turkey	3	[85,86,87]
Algeria	2	[88,89]
Kenya	2	[90,91]
Bangladesh	1	[92]
Cameroon	1	[93]
Cuba	1	[94]
Congo	1	[95]
Hungary	1	[96]
Jordan	1	[97]
Laos	1	[98]
Mexico	1	[99]
Mongolia	1	[100]
Peru	1	[101]
Philippines	1	[102]
Puerto Rico	1	[103]
Uganda	1	[104]
Zimbabwe	1	[105]
**Multi-Country Location**	**Number of HIAs**	**Reference**
Cameroon-Chad	2	[23,106]
Chile-Brazil-Mexico	1	[107]
Israel-India	1	[108]
Lithuania-Slovakia-Hungary-Bulgaria	1	[96]
Korea-Singapore-Viet Nam	1	[109]
101 countries across the globe	1	[110]

HIA: Health Impact Assessment.

**Table 3:** Number of studies by region and by topic.

HIA TOPIC	Asia	Africa	Europe/Middle East	Americas
Air Pollution (AP)	15	2	4	4
Construction	1	-	-	-
Development Project	1	5	-	1
Diabetes	1	-	1	-
Excreta management	1	-	-	-
Golden rice	1	-	-	-
Public & Green space	1	-	-	-
Urban Transport Planning	3	-	1	1
Vaccination	1	-	1	-
Homosexuality Bill	-	1	-	-
Infectious Diseases	-	1	-	-
Clinical Waste	-	1	-	-
Housing	-	-	2	-
Salt consumption	-	-	1	-
Cigarette smoking	-	-	-	2
Investment program	-	-	-	1
**Total number of studies**	**25**	**10**	**10**	**9**

**Table 4:** Number of studies by leading topic and by country.

Country	Air Pollution	Development Projects	Urban Transport Planning
Algeria	2		
Bangladesh	1		
China	11		1
India	1	1	1
Mongolia	1		
Thailand	2		
Iran	2		1
Turkey	2		1
Brazil	2		1
Chile-Brazil-Mexico	1		
Mexico	1		
Chad-Cameroon		2	
Zimbabwe		1	
Kenya		1	
Puerto Rico		1	
Laos		1	
Democratic Republic of Congo		1	

**Table 5:** Process evaluation results.

Process Evaluation Criteria	No. of Studies	Associated Factors	No. of Studies
Accessed baseline local data	57	Use of existing databases Primary data collection	43 14
Reported resources used	0	Open access to publication Reporting on HIA stages	40 17
Based on participatory approaches	7	Participatory stage Stakeholder profile	6 7
Considered multiple outcomes	53	Mortality outcomes Morbidity outcomes Social determinant outcomes Cost outcomes	33 43 11 17
Provided recommendation	35	Brief (as part of conclusion) Separate sections Data timing of delivery	29 6 7
Fostered cross-national collaboration	35	Local affiliation of first author	27

**Table 6:** No. of studies and countries by outcome.

No. Studies/No. of Countries	Mortality	Morbidity	SDH ^1^	Costs	Mortality Morbidity	Mortality Morbidity SDH	Mortality Morbidity SDH Costs
No. Studies	33	44	11	17	24	3	1
No. of Countries	15	25	13	11	12	3	1

^1^ Social Determinants of Health.

**Table 7:** Process Appraisal Checklist based on review and adapted from Parry and Kemm (2005) [48].

Stage	Prediction	Participation	Decision-Making	Resources
Screening	Clarify the issue at stake jointly with all parties Define the expected outcomes of HIA jointly with all parties	Conduct thorough stakeholder mapping Plan outreach strategy to stakeholders	Define the role of decision-makers in pushing HIA forward	Report on the costs of screening activities
Scoping	Define topic/sector of interest Scope for regions with similar features Identify local data sources and routinely collected data system Design HIA framework based on data type available and accessible data management technology	Approach institutions and individuals having access to adequate datasets	Define the decision makers agenda Fit the recommendations into adequate political timelines	Report on the costs of scoping activities
Appraisal	Adapt study area, indicators, and outcomes to increase validity and sensitivity of results	Report on technical working groups and workshops	Check whether involvement of decision-makers led to bias	Report on the costs needed to access the information needed
Dissemination	Craft clear and actionable recommendations	Deliver timely and compelling messages to appropriate audiences	Use multiple dissemination methods to access decision-makers	Report on the costs of activating dissemination process

## Supplementary Materials

The following are available online at https://www.mdpi.com/1660-4601/16/11/2018/s1, Supplementary S1: Protocol for systematic literature review; Supplementary S2: Extraction tool; Supplementary S3: List of studies.
