# Supplementary material for: Systematic Literature Review of Health Impact Assessments in Low and Middle-Income Countries
Source: Int J Environ Res Public Health. 2019 Jun 6;16(11):2018. doi: 10.3390/ijerph16112018 (PMC6603924; doi:10.3390/ijerph16112018)
Supplement: Supplementary file 1 [file ijerph-16-02018-s001.zip › ijerph-516995-Final_Supplementary/Supp1_FullProtocol_2805.docx]

**INFORMATION**

**Title:**

Systematic literature review of Health Impact Assessments in low and middle-income countries.

**Registration:**

PROSPERO Database since August 8, 2018

Systematic review registration number: crd420118102715

**Corresponding author:**

Mark Nieuwenhuijsen, Barcelona Institute for Global Health (ISGlobal), Barcelona Biomedical Research Park, Dr. Aiguader, 88; 08003, Barcelona, Spain. Tel +34 932147364; fax: +34932147301; E-mail address: [Mark.nieuwenhuijsen@isglobal.org](mailto:Mark.nieuwenhuijsen@isglobal.org)

**Support and Sponsors:**

Erasmus Mundus Joint Doctorate Program

Structured Summary

1. **Background**

There is no systematic review of HIA case studies in low income countries in the last 10 years. There is consensus that HIAs in developing countries are lacking but no study focuses on those that have been successfully conducted, and do not highlight their strengths and limitations.

1. **Objective**

The main objective of the review is to define and identify all HIA case studies that have been conducted in low income countries. The specific objectives are to (1) review the use of HIAs worldwide and retrieve those conducted in low income countries (2) describe what works, what doesn’t, in what types of countries, how, when and why; (3) define if there are differences between regions and countries in terms of use, types, experiences; (4) highlight strengths and limitations of HIA case studies and (5) propose recommendations forward for conducting HIA in low income countries.

1. **Data sources**

All case studies will be retrieved from pee-reviewed publications. Systematic database searches will be performed by two independent researchers and augmented by bibliographic review and expert consultation.

- 1. Electronic databases

1. Scopus
2. Medline
3. Web of Science
4. Sociological abstracts
5. LILACs
   1. Dates of coverage: No limit
   2. Search Strings

#1 Health Impact Assessment

health impact assessment/ OR (health impact* OR (health AND impact assess*)).ti,ab,kf.

#2 Emerging Markets and Developing Economies (IMF, 2016)

#3 study type

(scenario* or case or  policy or project* or program*).ti,ab,kf.

#4 extra study type

(city OR cities).ti,ab,kf.

- - 1. Example of a full search strategy in **Web of Science** in English

*Thomson Reuters, Web of Science Core Collection*

**#1 Health Impact Assessment**

TS=("health impact*" OR ("health" AND "impact assess*"))

**#2 Developing countries**

TS=("developing countr*" OR "low income countr*" OR "middle income countr*" OR "sub-sahara*" OR "subsahara*" OR "latin america" OR "caribbean" OR "south east asia" OR "southeast asia" OR "west indies" OR "antigua and barbuda" OR "kitts and nevis" OR "sao tome and principe" OR "st. lucia" OR "trinidad and tobago" OR "vincent and the grenadines" OR "afghanistan" OR "albania" OR "algeria" OR "angola" OR "argentina" OR "armenia" OR "azerbaijan" OR "bahamas" OR "bahrain" OR "bangladesh" OR "barbados" OR "belarus" OR "belize" OR "benin" OR "bhutan" OR "bolivia" OR "bosnia" OR "botswana" OR "brazil" OR "brunei" OR "bulgaria" OR "burkina faso" OR "burundi" OR "cabo verde" OR "cambodia" OR "cameroon" OR "central african republic" OR "chad" OR "chile" OR "china" OR "colombia" OR "comoros" OR "congo" OR "costa rica" OR "cote d'ivoire" OR "croatia" OR "cuba" OR "djibouti" OR "dominica" OR "dominican republic" OR "ecuador" OR "egypt" OR "el salvador" OR "eritrea" OR "ethiopia" OR "fiji" OR "georgia" OR "grenada" OR "guatemala" OR "guinea" OR "guyana" OR "haiti" OR "honduras" OR "hungary" OR "india" OR "indonesia" OR "iran" OR "iraq" OR "jamaica" OR "jordan" OR "kazakhstan" OR "kenya" OR "kiribati" OR "kosovo" OR "kuwait" OR "kyrgyz*" OR "lao" OR "lebanon" OR "lesotho" OR "liberia" OR "libya" OR "macedonia" OR "madagascar" OR "malawi" OR "malaysia" OR "maldives" OR "mali" OR "marshall islands" OR "mauritania" OR "mauritius" OR "mexico" OR "micronesia" OR "moldova" OR "mongolia" OR "montenegro" OR "morocco" OR "mozambique" OR "myanmar" OR "namibia" OR "nauru" OR "nepal" OR "nicaragua" OR "niger" OR "nigeria" OR "oman" OR "pakistan" OR "palau" OR "panama" OR "paraguay" OR "peru" OR "philippines" OR "poland" OR "puerto ric*" OR "qatar" OR "romania" OR "russia" OR "rwanda" OR "saint lucia" OR "samoa" OR "saudi arabia" OR "senegal" OR "serbia" OR "seychelles" OR "sierra leone" OR "solomon islands" OR "south africa" OR "south sudan" OR "sri lanka" OR "sudan" OR "suriname" OR "swaziland" OR "syria" OR "tajikistan" OR "tanzania" OR "thailand" OR "timor-leste" OR "togo" OR "tonga" OR "tunisia" OR "turkey" OR "turkmenistan" OR "tuvalu" OR "uganda" OR "ukraine" OR "arab emirates" OR "uruguay" OR "uzbekistan" OR "vanuatu" OR "venezuela" OR "vietnam" OR "yemen" OR "zambia" OR "zimbabwe")

**#3 study type (case studies)**

TS=("scenario*" OR "case" OR "policy" OR "project*" OR "program*")

**#4 Extra**

TS=("city" OR "cities")

**1 AND 2 AND 3 1.386 results**

Extra export: (1 AND 2 AND 4) NOT 3: 225 results

- - 1. Example of a full search strategy in **LILACS** in Spanish

*Literatura Latinoamericana y del Caribe en Ciencias de la Salud*

Link: <http://lilacs.bvsalud.org>

(mh:"Health Impact Assessment" OR ti:("health impact" OR "impacto en la salud" OR "impactos en la salud" OR "impacto sobre la salud" OR "impactos sobre la salud" OR "impacto na saude" OR "impactos na saude") OR ti:(health "impact assessment") OR (ti:("evaluacion del impacto" OR "evaluacion del impactos" OR "avaliacao do impacto" OR "avaliacao do impacto") ti:(salud OR saude))) OR (ab:("health impact" OR "impacto en la salud" OR "impactos en la salud" OR "impacto sobre la salud" OR "impactos sobre la salud" OR "impacto na saude" OR "impactos na saude") OR ab:(health "impact assessment") OR (ab:("evaluacion del impacto" OR "evaluacion del impactos" OR "avaliacao do impacto" OR "avaliacao do impacto") ab:(salud OR saude))) AND (tw:(scenario* OR case OR policy OR project* OR escenario* OR caso OR política OR proyecto* OR cenario* OR caso OR projeto* OR program*))

**Results: 265**

**Downloaded: 203**

**Cities**

((mh:"Health Impact Assessment" OR ti:("health impact" OR "impacto en la salud" OR "impactos en la salud" OR "impacto sobre la salud" OR "impactos sobre la salud" OR "impacto na saude" OR "impactos na saude") OR ti:(health "impact assessment") OR (ti:("evaluacion del impacto" OR "evaluacion del impactos" OR "avaliacao do impacto" OR "avaliacao do impacto") ti:(salud OR saude))) OR (ab:("health impact" OR "impacto en la salud" OR "impactos en la salud" OR "impacto sobre la salud" OR "impactos sobre la salud" OR "impacto na saude" OR "impactos na saude") OR ab:(health "impact assessment") OR (ab:("evaluacion del impacto" OR "evaluacion del impactos" OR "avaliacao do impacto" OR "avaliacao do impacto") ab:(salud OR saude))) AND (tw:(ciudad OR ciudades OR cidade OR cidades))) NOT (tw:(scenario* OR case OR policy OR project* OR escenario* OR caso OR política OR proyecto* OR cenario* OR caso OR projeto* OR program*))

**Results: 2**

1. **Study inclusion criteria, participants, and interventions**

General inclusion criteria

1. Individual HIA case studies only (original articles)
2. Published and peer reviewed papers only
3. In low and income countries only (the selection of the countries were based on the Emerging Markets and Developing Economies according to IMF’s definition (IMF, World Economic Outlook, 2016). EMDEs include 156 countries (see Table 1)
4. Following PICO table (see Table 2)

Specific inclusion criteria

1. The appraisal provides a comparison between different situations and brings an assessment that will change the status quo.
2. There is a clear statement and description of an intervention to be assessed. The intervention can be a programme, project or policy.
3. The intervention triggers a ‘before and after’ situation: it reports a change in the distribution of exposure for at least one health pathway.
4. The intervention addresses one or more problems in a specified population: it reports a change in at least one health outcome.
5. **Study appraisal and synthesis methods**

All methods applied PRISMA guidelines except for the meta-analysis section. The literature search, study selection, data extraction and synthesis lasted from May 13, 2018 to October 26, 2018. Appraisal was done by extracting general characteristics and by applying six process evaluation criteria:

- 1. Data items (List of variables for which data will be sought)

General characteristics:

Publication year, online accessibility, level at which conducted, intervention appraised, data type used, HIA self-identification, topic addressed.

Process evaluation criteria:

accessed baseline local data, reported resources used, based on participatory approaches, considered multiple outcomes, provided recommendation, fostered cross-national collaboration

- 1. Prioritization

We do not practice or prioritize based on a quality assessment.

- 1. Risk of bias in individual studies

Risk of bias will be assessed at study level sites.

- 1. Data Synthesis

No quantitative synthesis will be conducted.

- 1. Meta-Bias

This is not applicable.

1. **Results**

A search yielded 3178 records initially (excluding duplicates). After title screening (retaining 339 records) and abstract screening (resulting in 147 studies), we conducted full-text eligibility assessment and discarded 90 records not satisfying inclusion criteria. The final dataset included 57 studies(see Figure 1).

1. **Limitations; conclusions and implications of key findings;**

Limitations

Despite a solid systematic search, all relevant studies may not have been identified. The exclusion of grey literature may have induced publication bias as HIA in lower resource countries is frequently conducted by private and/or multilateral organisations (23,105,129). It is also possible that HIAs driven for specific interventions, on controversial topics and within tighter timelines were not made public or are restricted for use by particular individuals or institutions. Studies with negative findings, bad experiences or that were unsatisfactorily completed may have been less likely published. As another limitation, the criteria for evaluation were selected by authors’ professional judgement and may have impacted on the findings. The interpretation of evidence must also be done with care as they are mostly based on the subjective assessment of authors. While factors such as outcome calculation, regional distribution and level of implementation are objective to assess, the interpretation of other factors such as participation and recommendation were less evident. For instance, it was difficult to assess whether the absence of participation and recommendation were due to lack of reporting or lack of accomplishment.

Conclusions

The systematic review with focus on process evaluation of 57 case studies provided a unique opportunity for mapping and assessing HIA activity in LICs. There is an unequal distribution of HIAs in LICs. Studies from Asia lead in number and diversity of topics. The leading topics of HIA in LICs are air pollution, development projects and urban transport planning. Studies in Africa are significantly lagging behind in terms of first author affiliation. The process evaluation showed important variations the way HIAs are conducted and low uniformity in the reporting of six criterias. The limited reporting of resources used, weak participatory approaches and inconsistent delivery of recommendations were potential limitations to scaling HIA practice in LICs, while current opportunities to scaling HIAs are driven by access to local baseline data, the consideration of multiple outcomes and strong cross-national collaborations. Finally, this empirical auditing suggests that process evaluations are useful tools to assess what is needed to scale HIA practice to low-income countries and taking a step towards health for all.

Tables

**EMDE (IMF, World Economic Outlook, 2016)**

| \| Armenia \| \| --- \| \| Azerbaijan \| \| Belarus \| \| Georgia \| \| Kazakhstan \| \| Kyrgyz Republic \| \| Moldova \| \| Russia \| \| Tajikistan \| \| Turkmenistan \| \| Ukraine \| \| Uzbekistan \| \| Bangladesh \| \| Bhutan \| \| Brunei Darussalam \| \| Cambodia \| \| China \| \| Fiji \| \| India \| \| Indonesia \| \| Kiribati \| \| Lao P.D.R. \| \| Malaysia \| \| Maldives \| \| Marshall Islands \| \| Micronesia \| \| Mongolia \| \| Myanmar \| \| Nauru \| \| Nepal \| \| Palau \| | \| Papua New Guinea \| \| --- \| \| Philippines \| \| Samoa \| \| Solomon Islands \| \| Sri Lanka \| \| Thailand \| \| Timor-Leste \| \| Tonga \| \| Tuvalu \| \| Vanuatu \| \| Vietnam \| \| Albania \| \| Bosnia & Herzegovina \| \| Bulgaria \| \| Croatia \| \| Hungary \| \| Kosovo \| \| FYR Macedonia \| \| Montenegro \| \| Poland \| \| Romania \| \| Serbia \| \| Turkey \| \| Antigua and Barbuda \| \| Argentina \| \| The Bahamas \| \| Barbados \| \| Belize \| \| Bolivia \| \| Brazil \| \| Chile \| | \| Colombia  Costa Rica \| \| --- \| \| Cuba (added) \| \| Dominica \| \| Dominican Republic \| \| Ecuador \| \| El Salvador \| \| Grenada \| \| Guatemala \| \| Guyana \| \| Haiti \| \| Honduras \| \| Jamaica \| \| Mexico \| \| Nicaragua \| \| Panama \| \| Paraguay \| \| Peru \| \| Puerto Rico (added) \| \| St. Kitts and Nevis \| \| St. Lucia \| \| St. Vincent& Grenadines \| \| Suriname \| \| Trinidad and Tobago \| \| Uruguay \| \| Venezuela \| \| Afghanistan \| \| Algeria \| \| Bahrain \| \| Djibouti \| \| Egypt \| | \| Iran \| \| --- \| \| Iraq \| \| Jordan \| \| Kuwait \| \| Lebanon \| \| Libya \| \| Mauritania \| \| Morocco \| \| Oman \| \| Pakistan \| \| Qatar \| \| Saudi Arabia \| \| Sudan \| \| Syria \| \| Tunisia \| \| United Arab Emirates \| \| Yemen \| \| Angola \| \| Benin \| \| Botswana \| \| Burkina Faso \| \| Burundi \| \| Cabo Verde \| \| Cameroon \| \| Central African Republic \| \| Chad \| \| Comoros \| \| DR of the Congo \| \| Republic of Congo \| \| Côte d’ voire \| \| Equatorial Guinea \| | \| Eritrea \| \| --- \| \| Ethiopia \| \| Guinea \| \| Guinea-Bissau \| \| Kenya \| \| Lesotho \| \| Liberia \| \| Madagascar \| \| Malawi \| \| Mali \| \| M uritius \| \| Mozambique \| \| Namibia \| \| Niger \| \| Nigeria \| \| Rwanda \| \| São Tomé and Príncipe \| \| Senegal \| \| Seychelles \| \| Sierra Leo e \| \| South Africa \| \| South Sudan \| \| Swaziland \| \| Tanzania \| \| Togo \| \| Uganda \| \| Zambia \| \| Zimbabwe \| \|  \| |
| --- | --- | --- | --- | --- | --- | --- | --- | --- | --- | --- | --- | --- | --- | --- | --- | --- | --- | --- | --- | --- | --- | --- | --- | --- | --- | --- | --- | --- | --- | --- | --- | --- | --- | --- | --- | --- | --- | --- | --- | --- | --- | --- | --- | --- | --- | --- | --- | --- | --- | --- | --- | --- | --- | --- | --- | --- | --- | --- | --- | --- | --- | --- | --- | --- | --- | --- | --- | --- | --- | --- | --- | --- | --- | --- | --- | --- | --- | --- | --- | --- | --- | --- | --- | --- | --- | --- | --- | --- | --- | --- | --- | --- | --- | --- | --- | --- | --- | --- | --- | --- | --- | --- | --- | --- | --- | --- | --- | --- | --- | --- | --- | --- | --- | --- | --- | --- | --- | --- | --- | --- | --- | --- | --- | --- | --- | --- | --- | --- | --- | --- | --- | --- | --- | --- | --- | --- | --- | --- | --- | --- | --- | --- | --- | --- | --- | --- | --- | --- | --- | --- | --- | --- | --- | --- | --- | --- |

Table 1 List of EMDE countries

| Inclusion/Exclusion criteria according to PICO | | |
| --- | --- | --- |
|  | **Included** | **Excluded** |
| **Population** | All | N/A |
| **Intervention** | Health impact assessment at city, regional and national level | General and opinionated papers, HIA methodology |
| **Comparison** | N/A | N/A |
| **Study Design** | Case studies with qualitative and/or quantitative outcomes; and covering a all kind of policy sectors, projects, or programmes including topics such as housing, transport, regeneration and health. | Natural experiments, quasi-experimental studies, policy evaluations, observational studies, trials, protocols. HIA studies that have not been completed yet. |
| **Outcomes** | - All exposure pathways - All topics - Any sector - Any health outcomes - Affiliation of HIA practitioners - Complexity of HIAs - Duration of HIAs - Cost of HIAs | Outcomes not included in inclusion criteria. |
| **Publication type** | Peer reviewed journal article (original article) | Conference abstracts, conference presentations, study protocols, grey reports, dissertations, books, meetings |
| **Publication year** | No limit | No limit |
| **Setting** | EMDEs, Low or middle income country | Unspecified setting/population in high income country |
| **Language** | English, Portuguese, French, Spanish | All other languages |

Table 2 PICO table
